# Effects of Plant Genotype and Nitrogen Level on Bacterial Communities in Rice Shoots and Roots

**DOI:** 10.1264/jsme2.ME12212

**Published:** 2013-08-24

**Authors:** Kazuhiro Sasaki, Seishi Ikeda, Takashi Ohkubo, Chiharu Kisara, Tadashi Sato, Kiwamu Minamisawa

**Affiliations:** 1 Graduate School of Life Sciences, Tohoku University, 2–1–1 Katahira, Aoba-ku, Sendai 980–8577, Japan; 2 Memuro Research Station, National Agricultural Research Center for Hokkaido Region, Shinsei, Memuro-cho, Kasaigun, Hokkaido 082–0081, Japan

**Keywords:** automated ribosomal intergenic spacer analysis, nitrogen fertilization, microbial community structure, plant genotype, rice

## Abstract

To examine whether microbial community structure differs across rice genotypes, automated ribosomal intergenic spacer analysis (ARISA) was conducted. Nine cultivars of *Oryza sativa* ssp. *indica* or *japonica* and seven lines of other *Oryza* species were grown in paddy fields with low, standard, and high levels of N fertilization. Multidimensional scaling plots of bacterial ARISA for aerial parts of rice (shoots) revealed that the structure of shoot bacterial communities was significantly affected by plant genotype (*indica* or *japonica*) based on similarity tests, whereas root microbiomes were largely affected by the N fertilization level.

A wide range of microorganisms, including bacteria and fungi, have been found in the phyllosphere and rhizosphere (21, 25, 26), and reside in and on plants as endophytes and epiphytes (22, 25, 30). These symbiotic microbes could be important components of the proximate mechanisms underlying plant functional traits such as nutrient acquisition, plant defense, plant morphology and abiotic stress tolerance (7); however, many questions remain about the driving forces for shaping a community structure of plant-associated microbes (9).

In studies of how plant genetic factors control microbial community structure, artificially generated genetic variations are an important basic resource (11). In rice, Ikeda *et al.* (14) reported significant impacts of the *OsCCaMK* gene on the diversity of root-associated bacteria under both paddy and upland field conditions using *OsCCaMK* mutants screened from a *Tos17* mutant panel (2). *CCaMK* plays an important role in a common symbiosis pathway that leads to successful rhizobial and arbuscular mycorrhizal symbioses in plants (18). In addition, naturally occurring genetic variation in plants is another important resource (17). Genotypes derived from natural plant variations have been shown to affect root-associated bacterial communities in rice (10). Hardoim *et al.* (10) demonstrated that plant genotypes helped to shape bacterial community structures in rice roots; however, the effects of plant genotype on aerial parts of rice (shoot)-associated bacterial communities have not been analyzed by culture-independent methods.

Nitrogen is the most important mineral nutrient for crop production, and an adequate supply of nitrogen fertilizer is essential for sustaining high yields. Previous studies have shown that nitrogen application influences rice-associated microbial communities using polymorphisms of 16S rRNA and *nifH* genes (5, 15, 29).

In general, genetic fingerprinting techniques allow high throughput and comparative profiling of numerous samples for microbial community analyses (3, 26). Automated ribosomal intergenic spacer analysis (ARISA) is a highly sensitive method due to the laser detection of fluorescently labeled DNA, and it is a rapid and effective technique that can be used in conjunction with more accurate but labor-intensive methods (26). Using this method, Ikeda *et al.* (13) showed that the bacterial community structure in soybean roots could be classified into three groups according to the plant genotype.

In the present study, ARISA was used to reveal the effects of inter- and intraspecific genetic variations in the genus *Oryza* on bacterial communities. Nine cultivars of *O. sativa* and seven lines of other *Oryza* species were used for shoot-associated bacterial community analysis, and five cultivars of *O. sativa* were used for root-associated bacterial community analysis (Table 1).

A total of 48 seedlings of each cultivar and line were planted and grown in paddy fields at Kashimadai, Miyagi, Japan, as described by Obara *et al.* (24). Low nitrogen (LN) and standard nitrogen (SN) fields were managed with 0 and 30 kg N ha^−1^ of nitrogen fertilization, respectively. In the high nitrogen (HN) field, 270 kg N ha^−1^ of ammonium sulfate (Ube Agri-Materials, Tokyo, Japan) was applied in addition to basal fertilizer. To reach 270 kg N ha^−1^, 30 or 60 kg N ha^−1^ of ammonium sulfate was applied as additional fertilization every 2 weeks. Plants were sampled 90 days after transplanting. Each rice plant was dug up in a square (30 cm × 30 cm) to a depth of about 30 cm from the soil surface. The roots were washed with running tap water in the laboratory until the soil was removed from roots, and around 20 g shoots and a whole root in each plant were stored separately at −80°C until molecular analysis. A total of 20 g shoots and whole root tissue from one plant were separately ground to a powder in liquid nitrogen with a mortar and pestle. DNA was extracted from 200 to 300 mg powdered tissue by the DNA extraction method developed by Ikeda *et al.* (12). The remaining shoot samples were dried and then digested with sulfuric acid to quantify total nitrogen by the Kjeldahl method (Table S1).

The multidimensional scaling (MDS) method was used in conjunction with ARISA to evaluate similarities of bacterial communities in shoot and roots. The bacterial primer set ITSF/ITSReub (3) was used for ARISA. PCR amplification was carried out as described by Ikeda *et al.* (13). PCR products (1 μL) were mixed with 1 μL LIZ1200 internal size standard (Applied Biosystems, Foster City, CA, USA), and 20 μL deionized formamide was added. The mixture was denatured at 95°C for 5 min and cooled on ice. Next, the PCR product was placed in an ABI 3730xl DNA Analyzer (Applied Biosystems). The profile data (up to 1,200 bases) obtained by ARISA were initially analyzed with ABI GeneMapper software (Applied Biosystems) and were processed further with the RIBOSORT program (27) to assign fragment size and calculate the relative abundance of each ribotype. Fragments with fewer than 100 fluorescence units were eliminated from the analyses. Using the R program (27), an MDS plot was generated from the ribotypes using a similarity matrix produced by the RIBOSORT program with default parameters and the vegan package with the Bray-Curtis index of dissimilarity. Analyses of similarities (ANOSIM) were performed to test for significant differences using 1000 permutation tests. The resulting test statistic R indicates the degree of separation, ranging from 0 (no separation) to 1 (complete separation). Numbers of operational taxonomic units in each sample are shown in Table S2.

MDS was performed to compare the complex ARISA profiles of the bacterial community structures among the plant genotypes within the genus *Oryza* (Table 1). In *O. sativa*, MDS plots of the bacterial ARISA of shoot samples indicate that the bacterial communities were apparently separated between *japonica* and *indica* groups (Fig. 1A), which were statistically significant by similarity tests (ANO-SIM) (Table S3). MDS plots of the bacterial ARISA of shoot samples from other *Oryza* species (*O. glaberrima*, *O. rufipogon*, *O. punctata*, and *O. eichingeri*) (Table 1) did not form a distinct cluster, but were widely dispersed (Fig. 1A), suggesting that the diversity of bacterial community in shoots of *O. sativa* was lower than those of other *Oryza* species (Fig. 1A). This may reflect the presence of more genetic variations found in these *Oryza* species than in those of *O. sativa* (16).

MDS plots of the bacterial ARISA of shoot samples indicated that the community structures for *japonica* and *indica* genotypes formed clear clusters along dimension 1 under all nitrogen conditions (Fig. 2), suggesting that the effect of a plant genotype in *O. sativa* species on the shoot-associated bacterial community is greater than that of the nitrogen fertilization level. Although the nitrogen concentrations of rice shoots under HN conditions were apparently higher than those under other conditions (SN and LN), no correlation was observed between the N concentration and microbial community (Table S1).

Diverse environmental factors control the establishment of microbial communities in the phyllosphere, but recently it has been recognized that a plant genotype plays an important role in selecting phyllosphere communities (30). There are considerable variations between *indica* and *japonica* cultivars in the concentrations of chemical components, such as nitrate and heavy metals (1, 6). In addition, De Costa *et al.* (4) found that leaves from different rice genotypes have different culturable bacterial communities and concluded that the difference was significantly correlated with anatomical and physiological differences, such as leaf hair length and density, leaf temperature, stomatal density, and transpiration rate. Among these traits, the variation between *indica* and *japonica* cultivars in stomatal density and size and canopy temperature is well recognized, and the genetic factors underlying these traits have been identified (19, 28). Thus, these variations in the chemical characteristics and microstructures between *indica* and *japonica* might affect shoot-associated bacterial communities.

For root-associated bacterial community analysis, Sasanishiki, Taichung 65, Gemdjah Beton, IR24, and IR36 were examined (Table S2). MDS plots for bacterial ARISA of root samples indicated that root bacterial communities under SN and HN conditions formed a tight cluster (Fig. 1B), suggesting that the effect of nitrogen fertilization on the bacterial community associated with roots was greater than that of the plant genotypes examined. Demba Diallo *et al.* (5) showed that nitrogen treatment had a strong effect on the composition and diversity of expressed *nifH* pools that shifted towards methylotroph-related nitrogenases. In another study, colonization of sugarcane by *Acetobacter diazotrophicus* has been inhibited by high nitrogen fertilization (8). The bacterial community of bulk soil would help to understand the effect of nitrogen fertilization on the paddy field ecosystem (31).

Different genotypes of *Arabidopsis thaliana* have been used for structural comparisons of root microbial communities to improve our understanding of plant-microbe interactions (20). To the best of our knowledge, the present study is the first report that shoot-associated microbial communities are largely dependent on rice genotypes (*O. sativa* ssp. *japonica* and *indica*).

## Supplementary Material



## Figures and Tables

**Fig. 1:**
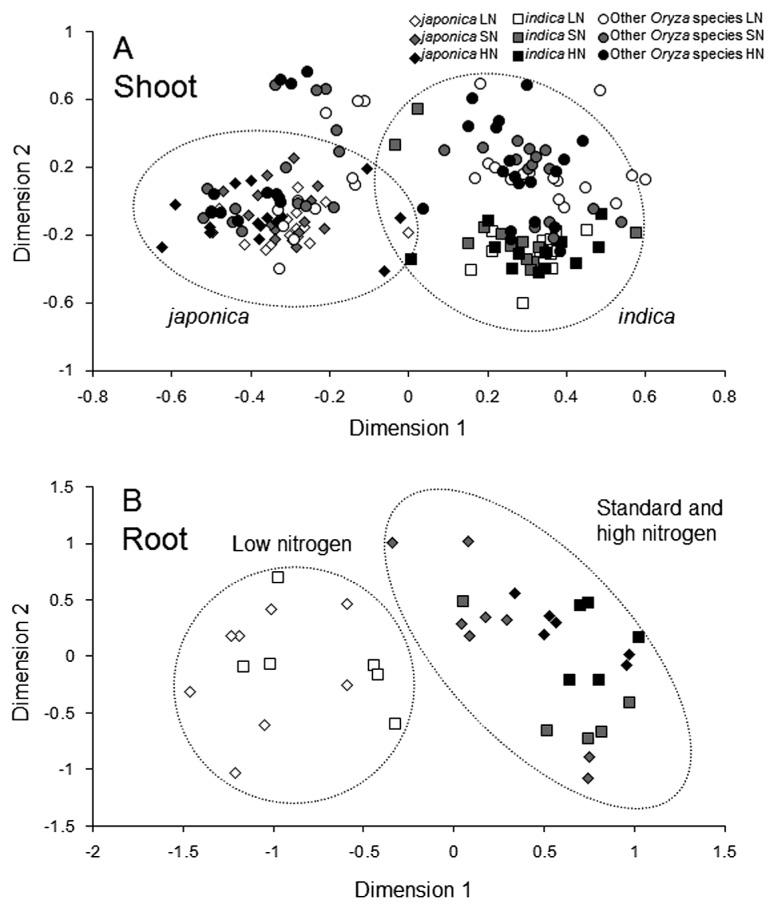
Multidimensional scaling plots generated from ARISA profiles with primers ITSF and ITSReub for rice (A) shoot- and (B) root-associated bacterial communities under low (LN), standard (SN), and high (HN) nitrogen conditions. Nine cultivars of *O. sativa* and seven lines of other *Oryza* species were used for shoot-associated bacterial community analysis with three replications, and five cultivars of *O. sativa* were used for root-associated bacterial community analysis with at least two replications (Table 1).

**Fig. 2:**
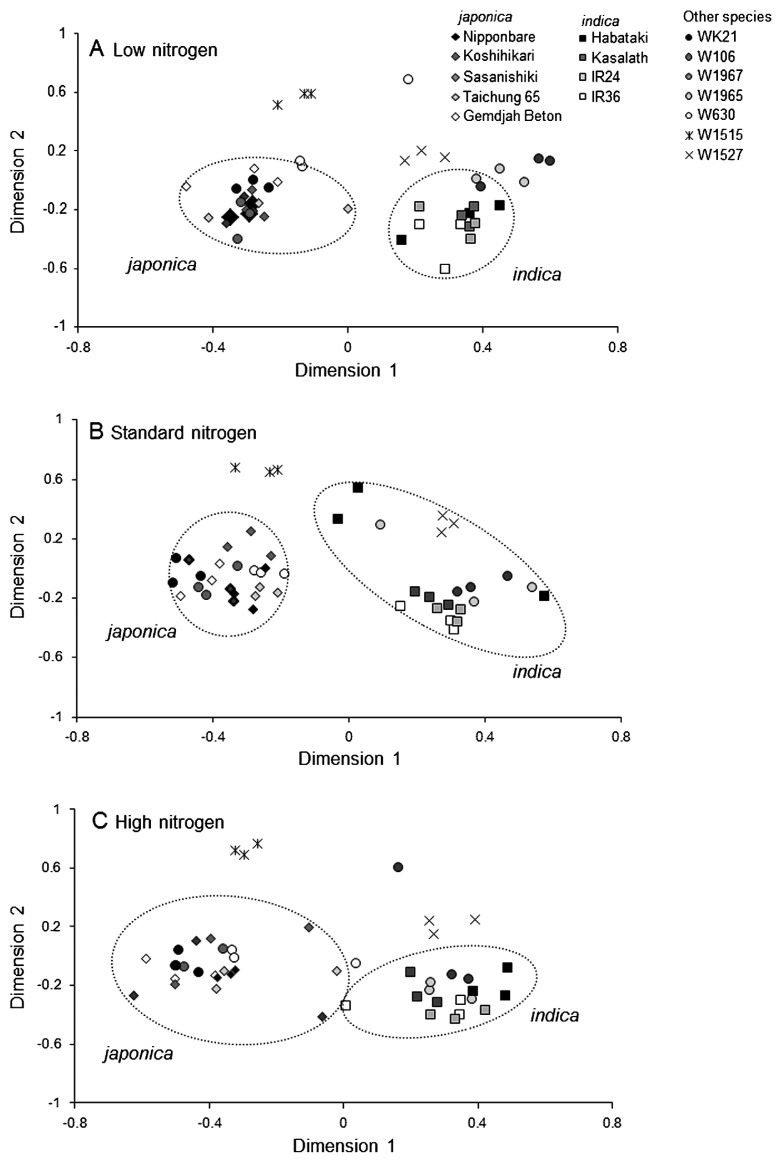
Multidimensional scaling plots generated from ARISA profiles with primers ITSF and ITSReub for rice shoot-associated bacterial communities under (A) low, (B) standard, and (C) high nitrogen conditions. *japonica* and *indica* indicate *O. sativa* spp. *japonica* and *indica*, respectively (Table 1). W106, W1967, W1965 and W630 belongs to *O. rufipogon* (Table 1) (*n*=3).

**Table 1:** Rice plant materials used in this study

Cultivar/line	Species	Genome^a^
Nipponbare	*O. sativa* L. ssp. *japonica*	AA
Sasanishiki	*O. sativa* L. ssp. *japonica*	AA
Taichung 65	*O. sativa* L. ssp. *japonica*	AA
Gemdjah Beton	*O. sativa* L. ssp. *japonica*	AA
Koshihikari	*O. sativa* L. ssp. *japonica*	AA
Habataki	*O. sativa* L. ssp. *indica*	AA
Kasalath	*O. sativa* L. ssp. *indica*	AA
IR24	*O. sativa* L. ssp. *indica*	AA
IR36	*O. sativa* L. ssp. *indica*	AA
WK21	*O. glaberrima* Steud.	AA
W106	*O. rufipogon* sensu lato	AA
W1965	*O. rufipogon* sensu lato	AA
W1967	*O. rufipogon* sensu lato	AA
W630	*O. rufipogon* sensu lato	AA
W1515	*O. punctata* Kotschy ex Steud.	BB
W1527	*O. eichingeri* Peter	CC

a Cultivars and lines were classified based on the genome composition according to the degree of meiotic chromosome pairing in hybrid plants (23).

## References

[1] Abe T, Taguchi-Shiobara F, Kojima Y, Ebitani T, Kuramata M, Yamamoto T, Yano M, Ishikawa S (2011). Detection of a QTL for accumulating Cd in rice that enables efficient Cd phytoextraction from soil. Breed Sci.

[2] Banba M, Gutjahr C, Miyao A, Hirochika H, Paszkowski U, Kouchi H, Imaizumi-Anraku H (2008). Divergence of evolutionary ways among common *sym* genes: CASTOR and CCaMK show functional conservation between two symbiosis systems and constitute the root of a common signaling pathway. Plant Cell Physiol.

[3] Cardinale M, Brusetti L, Quatrini P, Borin S, Puglia AM, Rizzi A, Zanardini E, Sorlini C, Corselli C, Daffonchio D (2004). Comparison of different primer sets for use in automated ribosomal intergenic spacer analysis of complex bacterial communities. Appl Environ Microbiol.

[4] De Costa DM, Rathnayake RMPS, De Costa WAJM, Kumari WMD, Dissanayake DMN (2006). Variation of phyllosphere microflora of different rice varieties in Sri Lanka and its relationship to leaf anatomical and physiological characters. J Agron Crop Sci.

[5] Demba Diallo M, Reinhold-Hurek B, Hurek T (2008). Evaluation of PCR primers for universal *nifH* gene targeting and for assessment of transcribed *nifH* pools in roots of *Oryza longistaminata* with and without low nitrogen input. FEMS Microbiol Ecol.

[6] Fan X, Jia L, Li Y, Smith SJ, Miller AJ, Shen Q (2007). Comparing nitrate storage and remobilization in two rice cultivars that differ in their nitrogen use efficiency. J Exp Bot.

[7] Friesen ML, Porter SS, Stark SC, von Wettberg EJ, Sachs JL, Martinez-Romero E (2011). Microbially mediated plant functional traits. Annu Rev Ecol Evol Syst.

[8] Fuentes-Ramírez LE, Caballero-Mellado J, Sepúlveda J, Martínez-Romero E (1999). Colonization of sugarcane by *Acetobacter diazotrophicus* is inhibited by high N-fertilization. FEMS Microbiol Ecol.

[9] Hardoim PR, van Overbeek LS, van Elsas JD (2008). Properties of bacterial endophytes and their proposed role in plant growth. Trends Microbiol.

[10] Hardoim PR, Andreote FD, Reinhold-Hurek B, Sessitsch A, van Overbeek LS, van Elsas JD (2011). Rice root-associated bacteria: insights into community structures across 10 cultivars. FEMS Microbiol Ecol.

[11] Hirochika H (2001). Contribution of the *Tos17* retrotransposon to rice functional genomics. Curr Opin Plant Biol.

[12] Ikeda S, Watanabe KN, Minamisawa K, Ytow N (2004). Evaluation of soil DNA from arable land in Japan using a modified direct-extraction method. Microbes Environ.

[13] Ikeda S, Rallos LEE, Okubo T, Eda S, Inaba S, Mitsui H, Minamisawa K (2008). Microbial community analysis of field-grown soybeans with different nodulation phenotypes. Appl Environ Microbiol.

[14] Ikeda S, Okubo T, Takeda N (2011). The genotype of the calcium/calmodulin-dependent protein kinase gene (*CCaMK*) determines bacterial community diversity in rice roots under paddy and upland field conditions. Appl Environ Microbiol.

[15] Knauth S, Hurek T, Brar D, Reinhold-Hurek B (2005). Influence of different *Oryza* cultivars on expression of *nifH* gene pools in roots of rice. Environ Microbiol.

[16] Kim H, Hurwitz B, Yu Y (2008). Construction, alignment and analysis of twelve framework physical maps that represent the ten genome types of the genus *Oryza*. Genome Biol.

[17] Koornneef M, Alonso-Blanco C, Vreugdenhil D (2004). Naturally occurring genetic variation in *Arabidopsis thaliana*. Annu Rev Plant Biol.

[18] Kouchi H, Imaizumi-Anraku H, Hayashi M, Hakoyama T, Nakagawa T, Umehara Y, Suganuma N, Kawaguchi M (2010). How many peas in a pod? Legume genes responsible for mutualistic symbioses underground. Plant Cell Physiol.

[19] Laza MRC, Kondo M, Ideta O, Barlaan E, Imbe T (2011). Quantitative trait loci for stomatal density and size in lowland rice. Euphytica.

[20] Lundberg DS, Lebeis SL, Paredes SH (2012). Defining the core *Arabidopsis thaliana* root microbiome. Nature.

[21] Mano H, Morisaki H (2008). Endophytic bacteria in the rice plant. Microbes Environ.

[22] Mano H, Tanaka F, Nakamura C, Kaga H, Morisaki H (2007). Culturable endophytic bacterial flora of the maturing leaves and roots of rice plants (*Oryza sativa*) cultivated in a paddy field. Microbes Environ.

[23] Nonomura KI, Morishima H, Miyabayashi T, Yamaki S, Eiguchi M, Kubo T, Kurata N (2010). The wild *Oryza* collection in National BioResource Project (NBRP) of Japan: history, biodiversity and utility. Breed Sci.

[24] Obara M, Sato T, Sasaki S, Kashiba K, Nagano A, Nakamura I, Ebitani T, Yano M, Yamaya T (2004). Identification and characterization of a QTL on chromosome 2 for cytosolic glutamine synthetase content and panicle number in rice. Theor Appl Genet.

[25] Rosenblueth M, Martínez-Romero E (2006). Bacterial endophytes and their interactions with hosts. Mol Plant Microbe Interact.

[26] Saito A, Ikeda S, Ezura H, Minamisawa K (2007). Microbial community analysis of the phytosphere using culture-independent methodologies. Microbes Environ.

[27] Scallan U, Liliensiek A, Clipson N, Connolly J (2008). RIBOSORT: a program for automated data preparation and exploratory analysis of microbial community fingerprints. Mol Ecol Res.

[28] Takai T, Yano M, Yamamoto T (2010). Canopy temperature on clear and cloudy days can be used to estimate varietal differences in stomatal conductance in rice. Field Crops Res.

[29] Tan Z, Hurek T, Reinhold-Hurek B (2003). Effect of N-fertilization, plant genotype and environmental conditions on *nifH* gene pools in roots of rice. Environ Microbiol.

[30] Whipps JM, Hand P, Pink D, Bending GD (2008). Phyllosphere microbiology with special reference to diversity and plant genotype. J Appl Microbiol.

[31] Wu L, Ma K, Li Q, Ke X, Lu Y (2009). Composition of archaeal community in a paddy field as affected by rice cultivar and N fertilizer. Microb Ecol.

