# Supplementary material for: Effects of Plant Genotype and Nitrogen Level on Bacterial Communities in Rice Shoots and Roots
Source: Microbes Environ. 2013 Aug 24;28(3):391–5. doi: 10.1264/jsme2.ME12212 (PMC4070954; doi:10.1264/jsme2.ME12212)
Supplement: Supplementary file 1 [file 28_391_s1.pdf]

Supplementary materials

# Effects of plant genotype and nitrogen level on bacterial communities in rice shoot and root

KAZUHIRO SASAKI[1,2], SEISHI IKEDA[1,3], TAKASHI OHKUBO[1], CHIHARU KISARA[1], TADASHI SATO[1], and KIWAMU MINAMISAWA[1,*]

[1]*Graduate School of Life Sciences, Tohoku University, 2-1-1 Katahira, Aoba-ku, Sendai 980-8577, Japan*

[2] Present address: *Plant Breeding, Genetics, and Biotechnology, International Rice Research Institute, DAPO Box 7777, Metro Manila, The Philippines*

[3]*Memuro Research Station, National Agricultural Research Center for Hokkaido Region, Shinsei, Memuro-cho, Kasaigun, Hokkaido 082-0081, Japan*

Table S1. Nitrogen concentration (%) of shoot cultivated in the paddy fields under low (LN), standard (SN) and high nitrogen (HN) condition

| Cultivar/line | LN | SN | HN |
|---|---|---|---|
| Nipponbare | 0.7 ± 0.1[a] | 0.9 ± 0.1 | 1.3 ± 0.2 |
| Sasanishiki | 0.8 ± 0.2 | 0.5 ± 0.0 | 1.4 ± 0.2 |
| Taichung 65 | 0.9 ± 0.2 | 0.7 ± 0.0 | 1.8 ± 0.1 |
| Germdjah Benton | 1.0 ± 0.2 | 0.8 ± 0.0 | 1.7 ± 0.2 |
| Koshihikari | 0.8 ± 0.1 | 0.7 ± 0.0 | 1.4 ± 0.1 |
| Habataki | 0.8 ± 0.1 | 0.6 ± 0.1 | 1.7 ± 0.1 |
| Kasalath | 0.6 ± 0.1 | 0.6 ± 0.1 | 1.6 ± 0.2 |
| IR24 | 0.9 ± 0.1 | 0.8 ± 0.2 | 1.5 ± 0.3 |
| IR36 | 0.8 ± 0.2 | 0.7 ± 0.1 | 1.9 ± 0.3 |
| WK21 | 0.8 ± 0.1 | 0.7 ± 0.1 | 1.7 ± 0.3 |
| W106 | 0.9 ± 0.1 | 1.2 ± 0.2 | 2.0 ± 0.1 |
| W1965 | 1.1 ± 0.1 | 1.1 ± 0.1 | 1.8 ± 0.2 |
| W1967 | 0.9 ± 0.1 | 0.7 ± 0.0 | 1.4 ± 0.1 |
| W630 | 1.3 ± 0.1 | 1.2 ± 0.2 | 1.7 ± 0.1 |
| W1515 | 0.7 ± 0.1 | 0.6 ± 0.0 | 1.3 ± 0.0 |
| W1527 | 1.2 ± 0.3 | 1.1 ± 0.1 | 1.8 ± 0.2 |

a Standard deviation (*n*=3)

Table S2. Number of operational taxonomic unit in an ARISA community profile

| Cultivar/line | Shoot LN | Shoot SN | Shoot HN | Root LN | Root SN | Root HN |
|---|---|---|---|---|---|---|
| Nipponbare | 89.3 ± 26.0 [a] | 97.0 ± 1.0 | 74.0 ± 15.7 | | | |
| Sasanishiki | 99.0 ± 6.1 | 168.7 ± 63.1 | 68.0 ± 12.1 | 49.0 ± 16.5 | 54.0 ± 7.5 | 29.0 |
| Taichung 65 | 72.0 ± 20.1 | 130.0 ± 20.3 | 85.3 ± 10.0 | 51.0 | 51.3 ± 12.9 | 38.0 |
| Germdjah Benton | 62.3 ± 6.7 | 60.7 ± 12.3 | 80.7 ± 28.4 | 14.7 ± 13.2 | 39.0 | 37.5 |
| Koshihikari | 79.7 ± 30.7 | 83.7 ± 20.0 | 37.0 ± 29.2 | | | |
| Habataki | 81.7 ± 13.5 | 99.3 ± 9.7 | 89.0 ± 6.2 | | | |
| Kasalath | 53.7 ± 6.7 | 67.3 ± 5.9 | 71.0 ± 13.5 | | | |
| IR24 | 72.0 ± 27.9 | 67.0 ± 7.5 | 59.7 ± 10.5 | 25.3 ± 8.1 | 30.5 | 33.3 ± 15.0 |
| IR36 | 64.0 ± 7.8 | 80.7 ± 11.0 | 51.3 ± 9.1 | 37.3 ± 6.7 | 38.3 ± 7.8 | 38.5 |
| WK21 | 128.3 ± 23.2 | 76.7 ± 15.9 | 59.3 ± 12.9 | | | |
| W106 | 117.3 ± 12.1 | 92.3 ± 5.1 | 77.3 ± 18.8 | | | |
| W1965 | 110.3 ± 11.7 | 80.7 ± 5.1 | 73.0 ± 30.6 | | | |
| W1967 | 120.0 ± 26.1 | 69.0 ± 14.0 | 46.0 ± 12.1 | | | |
| W630 | 184.3 ± 19.1 | 91.0 ± 5.3 | 81.7 ± 14.5 | | | |
| W1515 | 66.0 ± 15.4 | 61.3 ± 11.9 | 47.7 ± 23.2 | | | |
| W1527 | 142.0 ± 22.1 | 77.7 ± 17.0 | 55.3 ± 9.8 | | | |

[a] Standard deviation (n=3)

Table S3. Analysis of similarity (ANOSIM) tests of pairwise compariosn between rice plant genotypes.

| Pairwise comparison | | *R*-statistic | *P*-value |
|---|---|---|---|
| *japonica* | *indica* | 0.84 | 0.001 |
| *japonica* | Other *Oryza* species | 0.16 | 0.001 |
| *indica* | Other *Oryza* species | 0.21 | 0.001 |

See text for the method and paiewise combinations of ANOSIM anaysis
